# Paralytic Ileus as an Extra-Pulmonary Manifestation of COVID-19

**DOI:** 10.7759/cureus.35480

**Published:** 2023-02-26

**Authors:** Navid Moghimi, Rasmus D Bojesen, Kenneth Rütz

**Affiliations:** 1 Gastrointestinal Surgery, Zealand University Hospital, Koge, DNK; 2 Gastrointestinal Surgery, Slagelse Hospital, Slagelse, DNK

**Keywords:** covid-19 ards, paralytic ileus, sars-cov-2 and covid-19, gastrointestinal ileus, covid 19

## Abstract

Extra-pulmonary manifestations of COVID-19 (SARS-CoV-2) are of increasing interest as a consequence of the increase in cases worldwide and a better understanding of the pathophysiology of the disease. However, gastrointestinal symptoms are rarely described but are a common occurrence.

We report a case of a 62-year-old male with severe pulmonary infection with COVID-19, who presented with abdominal pain, hematemesis, bloody diarrhea, and abdominal distention, which led to the diagnosis of paralytic ileus after diagnostic laparoscopy. Further, we discuss the potential pathophysiological mechanisms behind this manifestation of COVID-19.

## Introduction

COVID-19 was initially presented and thought of as primarily an infectious respiratory disease, typically presenting with pulmonary symptoms such as cough and sore throat that can progress to COVID‐19 pneumonia and acute respiratory distress syndrome (ARDS) [1]. However, with the spread of the pandemic and the increase in insight into the disease pathophysiology, many extra-pulmonary manifestations are being recognized by researchers and clinicians. These include thrombosis, hyperglycemia, cardiac, renal, hepatic, neurological, ocular, dermatological, and gastrointestinal symptoms. Gastrointestinal symptoms may be present in up to 26% of patients [2]. The most common gastrointestinal symptoms, including diarrhea, abdominal pain, loss of appetite, vomiting, and anorexia, are frequently observed in patients with COVID-19 infection [3]. Abnormal liver function is often found in patients with COVID-19, and some studies have shown that fecal shedding of the virus has also been observed [4]. We report a case of COVID‐19 with severe pulmonary infection who presented to our Emergency department with abdominal pain, bloody diarrhea, and paralytic ileus as gastrointestinal manifestations of COVID-19.

## Case presentation

A 62-year-old male patient was admitted to our center in severe respiratory distress after seven days of fever, cough, shortness of breath, and loss of taste and smell. He complained of severe acute peri-umbilical pain, which had arisen a few hours before admission. Abdominal pain is described as progressively worsening, non-radiating and acute in onset.

The patient also described an episode of bloody diarrhea, which occurred 1.5 days before admission, and 2 to 3 episodes of hematemesis. The patient was an active smoker (1 pack/day) and known to have hypertension and mild COPD for many years, but no previous surgeries and no history of coagulopathies.

**Figure 1:**
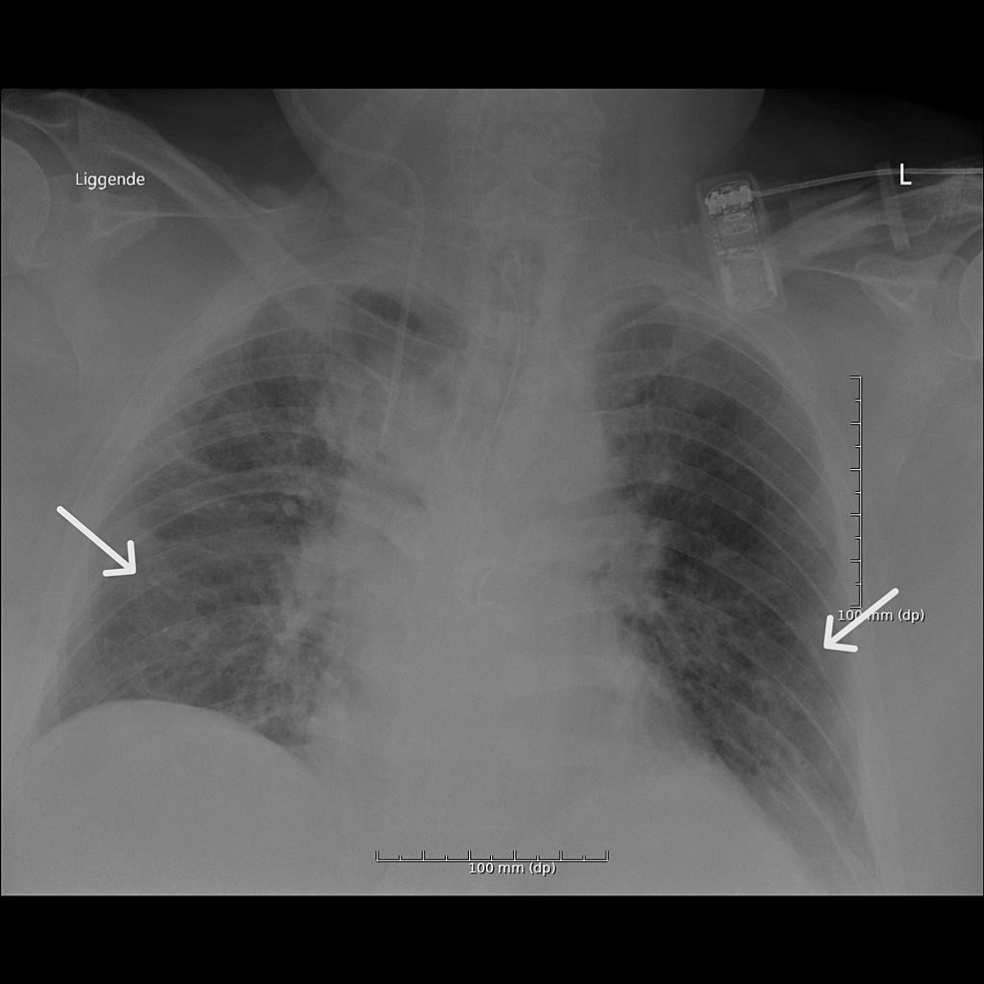
Chest X-ray on the day of admission showed bilateral patchy opacities.

**Figure 2:**
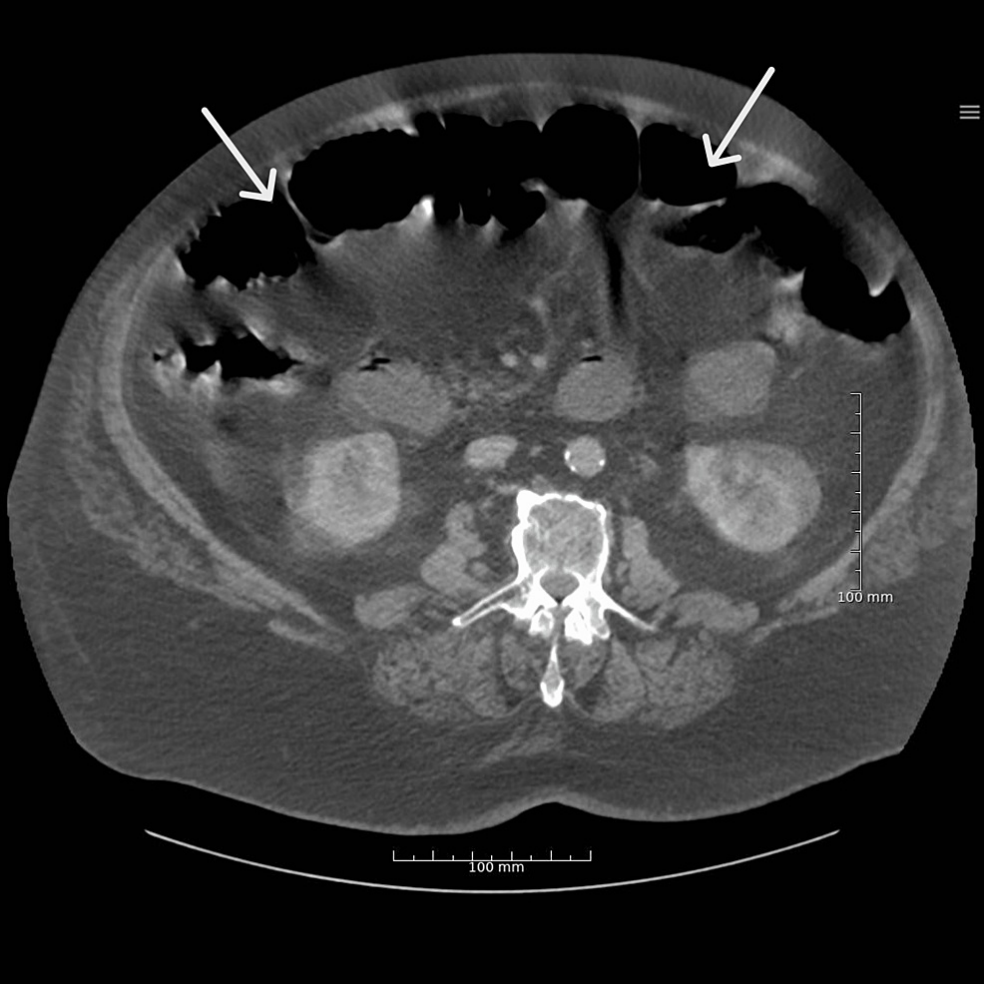
CT abdomen showing small bowel obstruction (SBO) with dilated loops of the small bowel.

**Figure 3:**
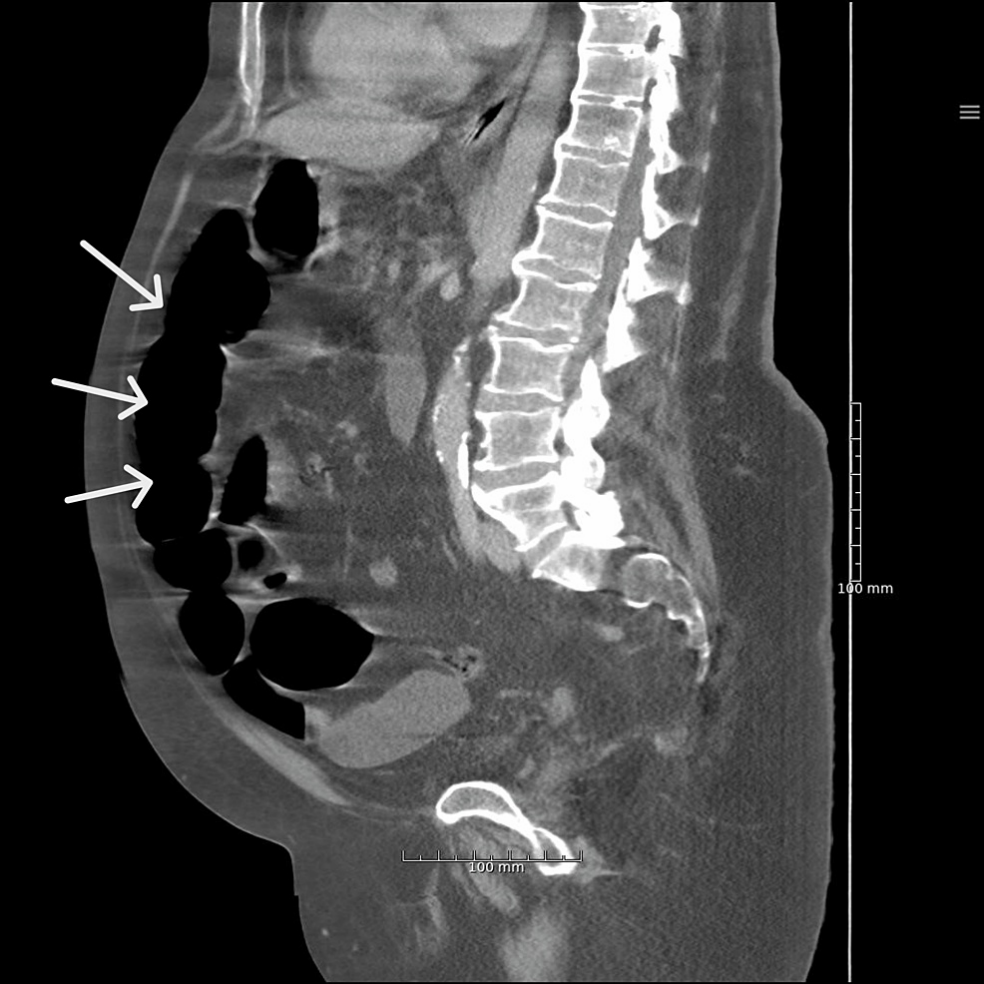
CT abdomen showing small bowel obstruction (SBO) with dilated loops of the small bowel.

The patient was hemodynamically not stable with a blood pressure of 90/50 mmHg, heart rate of 150 beats per minute, respiratory frequency of 46 per minute, a temperature of 36°C, and oxygen saturation of 92% without supplementation. On physical examination, he was in mild distress from the abdominal pain and had a distended abdomen with diffuse tenderness and sluggish bowel sounds, without rebound tenderness or guarding. Initial blood work found elevated lactate dehydrogenase (LDH) and C-reactive protein and showed leukocytosis with a normal coagulation profile. His oropharyngeal swab using PCR was COVID-19 positive. Chest X-ray showed bilateral patchy opacities (Figure 1). An abdominal CT scan was conducted, which showed signs of small bowel obstruction, with significant diffusely distended bowel and resolution of small bowel dilatation (Figures 2, 3).

The patient was evaluated by the surgical team and was rushed to the operating room for a diagnostic laparoscopy. During surgery, the small bowel had significant dilation and was without vascular congestion, obstruction, or perforation. No resection was required, and the surgery was concluded at this point. The patient tolerated the operation well, without complications, and COVID-19-related paralytic small bowel ileus was deemed as the most likely diagnosis. The patient was admitted to the intensive care unit with a case of severe COVID-19 pneumonia. He was extubated on day four of admission. Following the return of regular bowel function on postoperative day five, he was discharged from surgical consulting services. No further episodes of hematochezia or hematemesis were observed during admission. 

## Discussion

COVID-19 primarily manifests with pulmonary symptoms such as pneumonia, bronchitis, and acute respiratory distress syndrome (ARDS). In addition, extra-pulmonary complications are common, and some studies have shown that gastrointestinal symptoms can manifest even before the onset of typical respiratory symptoms [1]. Further, critically ill patients with COVID-19 may develop gastrointestinal complications during their hospital stay, including bowel ischemia, gastrointestinal bleeding, pancreatitis, Ogilvie syndrome, and severe ileus [5]. There seems to be an association between GI symptoms and the severity of illness [5], but whether the high incidence of gastrointestinal complications is a manifestation of critical illness in general or is specific to COVID-19 remains unclear.

The pathophysiology of gastrointestinal damage in COVID-19 is probably multifactorial. Infection with SARS-CoV-2 occurs when the virus enters cells by binding to the angiotensin‐converting enzyme‐2 (ACE-2) receptor [6] and/or receptors for transmembrane protease serine 2 (TMRPSS2) [7] on cell surfaces. Both of these receptors are expressed by the lungs but are also concomitantly expressed in the small intestine [8]. Thus, the ACE-2 receptors and the TMRPSS2 receptors along the epithelial lining of the gut can act as host-cell receptors for SARS-CoV-2 and thereby explain a direct involvement of the small intestine and other abdominal organs [9,10]. Several studies have found that SARS-CoV-2 RNA can be found in stool in approximately 50% of patients with active infection [11], indicating the presence of viral infection along the GI tract. The local presence of SARS-CoV-2 along the GI tract is associated with greater intestinal inflammation and the release of cytokines such as interleukin-6 (IL-6) and Tumor Necrosis Factor-α [12]. These cytokines play an important role in causing the local and systemic inflammatory response and are released in response to tissue injury and pro-inflammatory stimulus from the innate and adaptive immune system. Thus, IL-6 and TNF-α concentrations are elevated after various conditions such as surgery, trauma, sepsis, and critical illness and are central gatekeepers of the ‘cytokine storm’ seen in cases of severe COVID-19 infection. High IL-6 levels are associated with postoperative- [13] and sepsis-induced paralytic ileus. Thus, it is not clear if the gastrointestinal symptoms related to COVID-19 are a consequence of the systemic release of cytokines due to local infection or a combination of both. However, a specific consequence of severe infection with COVID-19 and subsequent release of cytokines are local submucosal endotheliitis [6], which leads to endothelial damage and micro ischemic damage of the GI tract [7]. Further, severe COVID-19 infections are associated with coagulopathies and fibrinolysis [14], which also may contribute to gastrointestinal symptoms such as abdominal pain, diarrhea, paralytic ileus, and hematochezia.

## Conclusions

Gastrointestinal manifestations of COVID-19 infection may pre-exist and not necessarily be accompanied by respiratory symptoms, and paralytic ileus should be considered as a possible presentation of COVID-19 infection that necessitates timely diagnosis and management.
